# Correlations of Host Genetics and Gut Microbiome Composition

**DOI:** 10.3389/fmicb.2016.01357

**Published:** 2016-08-30

**Authors:** Krystyna Dąbrowska, Wojciech Witkiewicz

**Affiliations:** ^1^Research and Development Center, Regional Specialized HospitalWrocław, Poland; ^2^Bacteriophage Laboratory, Institute of Immunology and Experimental Therapy, Polish Academy of SciencesWrocław, Poland

**Keywords:** microbiome, gene polymorphism, QTL, personalized medicine, gut, bacteria

## Abstract

The human gut microbiome has a considerable impact on host health. The long list of microbiome-related health disorders raises the question of what in fact determines microbiome composition. In this review we sought to understand how the host itself impacts the structure of the gut microbiota population, specifically by correlations of host genetics and gut microbiome composition. Host genetic profile has been linked to differences in microbiome composition, thus suggesting that host genetics can shape the gut microbiome of the host. However, cause-consequence mechanisms behind these links are still unclear. A survey of the possible mechanisms allowing host genetics to shape microbiota composition in the gut demonstrated the major role of metabolic functions and the immune system. A considerable impact of other factors, such as diet, may outweigh the effects of host genetic background. More studies are necessary for good understanding of the relations between the host genetic profile, gut microbiome composition, and host health. According to the idea of personalized medicine, patient-tailored management of microbiota content remains a fascinating area for further inquiry.

## Introduction

The human gut houses a complex community of microbes whose number is estimated to be 10 times higher than the number of cells in the whole body. These microbial communities, called the gut microbiome, are dynamic populations that differ from one person to another and that change their structure with time. Gut microbiotas interact with their hosts in many ways; thus composition of a microbiome may impact the balance of the whole system, and changes in microbial communities may exert significant effects on an individual’s health. Thus, microbes can be used in strategies addressing modulation of microbiome functionality, i.e., they can be used as probiotics (Savage, 1977; Kau et al., 2011; Linares et al., 2016). An extensive review on probiotic roles played by the microbiota has recently been published by Linares et al. (2016).

The importance of the gut microbiota for human health has been widely appreciated during this and the previous decade. The least surprising was their effect on gastrointestinal tract functions and diseases such as ulcerative colitis (Macfarlane et al., 2005; Sokol et al., 2006a,b; Linares et al., 2016) gastroenteritis (Barman et al., 2008), and celiac disease (Nadal et al., 2007). Abnormalities in composition of gut bacteria are considered as an important factor promoting inflammatory bowel disease including Crohn’s disease (Kassinen et al., 2007; Dicksved et al., 2008; Collins et al., 2009; Linares et al., 2016; Manuc et al., 2016). Since gut bacteria are involved in metabolic transformations and energy harvest, they have been reported as a biotic factor regulating body weight, potentially linked to a risk of obesity and other metabolic disorders (Backhed et al., 2004; Turnbaugh et al., 2006, 2009; Cani and Delzenne, 2009; Ley, 2010). However, in this field also controversies have been pointed out, like the complex character of studied phenomenon, the necessity of studies based on observation and description in opposition to studies performed to confirm a hypothesis and the independence from the food industry (Angelakis et al., 2012; Lagier et al., 2012).

Gut microbiota composition has been linked to functions of organs and tissues far beyond the gut itself. Probably the most spectacular is the so-called gut–brain axis; biochemical signaling between the gastrointestinal tract and the nervous system is important for healthy brain function. This relationship involves the gut microbiome, the composition of which may be linked to neuropsychiatric diseases (Mu et al., 2016; Yarandi et al., 2016). Another important role of the natural microbiome is liver homeostasis, since bacterial metabolites in dysbiosis can be linked to the pathogenesis of liver disease (Haque and Barritt, 2016). Microbial metabolites belonging to short-chain fatty acids (butyrate) may affect the whole system of the host, even mitigating graft-versus-host disease (GVHD), the most probably by regulation of histone acetylation (Mathewson et al., 2016). Some findings reveal that the microbiomes of the lung and gut contribute to the pathogenesis of asthma and allergy by regulation of helper T cell subsets that affect the development of immune tolerance (Russell et al., 2012; Riiser, 2015).

The long list of microbiome-related health disorders raises the question of what in fact decides on microbiome composition. Ecological sciences define factors that shape microbial community structure as a combination of environmental factors such as diet, and host-defined ones. In fact, there is a high interaction between the microbiome and the host, and for that reason both of them have evolved together, which may explain possible microbiome adaptations (Cavender-Bares et al., 2009; Walter and Ley, 2011; Leamy et al., 2014; Linares et al., 2016). Relating individual microbiome composition to host genetics may constitute a link between probiotic studies and personalized medicine. In this review we aim to bring together recent findings that demonstrate the link between human host genetics and the gut microbiota, from the perspective of practical implications of this knowledge for humans’ health.

## Links Between Host Genetic Profile and Individual Gut Microbiome in Humans

The question of how genotype and environmental exposure influence the gut microbiome has been addressed in a twin pairs study by Turnbaugh et al. (2009). Fecal microbiota were characterized in 154 adult individuals comprising female monozygotic or dizygotic twins and their mothers, if available. In this group, the gut microbiota was similar among family members, but individual variations were observed, i.e., specific bacterial lineages were present in each person’s gut. These variations were assessed in monozygotic and in dizygotic twins, and the analysis showed a comparable degree of co-variation between these groups. The authors found their observation consistent with an earlier study of adult twins by fingerprinting (Zoetandal et al., 2001). These findings contradict the hypothesis of a substantial impact of host genetics on microbiome composition and they are in line with observation of Murphy et al. (2015) who studied dichorionic triplet sets. In these group only at the 1st month of life monozygotic pair shared microbiota distinct to the fraternal sibling. At the 12th month no significant differences were observed. However, some limitations of the studied have been pointed out. First, small groups: 20–30 twin pairs in each category in studies of Turnbaugh et al. (2009) and three triplet sets in studies of Murphy et al. (2015). Second, broad measures of the microbiome composition instead of individual bacterial representation was investigated (Davenport et al., 2015; Murphy et al., 2015).

Recent studies by Goodrich et al. (2014) analyzed microbiotas in fecal samples obtained from the TwinsUK population (416 twin pairs); microbiomes were found more similar for monozygotic than dizygotic twins. The study also revealed differences between bacterial families; the analyses of distance metrics in the three most dominant bacterial families – the *Lachnospiraceae* and *Ruminococcaceae* (*Firmicutes*) and *Bacteroidaceae* – demonstrated greater similarities between monozygotic twins within the *Ruminococcaceae* and *Lachnospiraceae* than those between dizygotic twins. Similar pair-wise diversity was restricted to the *Bacteroidaceae* family, so this group was suggested to be more responsive to environmental factors (Goodrich et al., 2014). Moreover, datasets from Turnbaugh et al. (2009) and Yatsunenko et al. (2012) were re-analyzed, validating the observation that the representation of bacterial taxa in the gut is more similar within monozygotic than dizygotic twin pairs (Goodrich et al., 2014).

Davenport et al. (2015) applied a genome-wide association study (GWAS) to investigate the fecal microbiome from a religious isolated group, the Hutterites who live on communal farms. The Hutterites are not an outbred population, but rather a genetic isolate exhibiting a strong founder effect (Coghlan and Zelinski, 2016). They are also an isolated population due to the communal farming, therefore variations of environmental factors have less impact on individuals’ microbiomes (Davenport et al., 2015; Igartua et al., 2016). These features make this population a unique model for genome and microbiome studies. The study in 127 participants demonstrated that the abundances of at least eight bacterial taxa were associated with host genome single nucleotide polymorphisms (SNPs), including those previously associated with BMI in obesity studies. The differentiation of bacterial taxa abundances between male and female participants was also reported, however, this element must be considered with caution. Hutterite society practice substantially different daily activities of men and women which could drive sex-specific differences (Davenport et al., 2015; Davenport, 2016). In the report within the Human Microbiome Project, most variation in the human microbiome could not be well explained by their relation to gender (Human Microbiome Project Consortium, 2012a,b).

## Murine Models Demonstrate that Host Genetic Profile Can Shape Gut Microbiome

The relation between the composition of gut microbiota and the host genetic profile has been clearly demonstrated in murine models. Benson et al. (2010) showed that composition of the gut microbiota behaves as a polygenic trait (i.e., resultative phenotype cumulates effects of more than one gene), and they identified in mice 18 host quantitative trait loci (QTL) that correlated with relative abundances of particular microbial groups. This correlation showed that heritable genetic factors may govern intimate associations between the host and its microbiota, although additional efforts will be needed to explain in details which physiological mechanisms are involved. The study was done in a large (*n* = 645) murine intercross model (*G*_4_) in which the environmental factors were carefully controlled (**Table 1**).

**Table 1:** Correlations of body traits and microbiome traits by the same quantitative trait loci.

Correlation by QTLs		Reference
Body trait	Microbiome trait	
Fat content	OTU3615 (Actinobacteria)	Leamy et al., 2014
Fat content	OTU22207 (Alistepes)	Leamy et al., 2014
Weight and fat content	OTU30840 (Clostridium)	Leamy et al., 2014
Weight	*Lactococcus lactis*	Leamy et al., 2014
Immune response	Coriobacteriaceae	Benson et al., 2010
Immune response	*Lactococcus*	Benson et al., 2010
Susceptibility to colon tumors	Coriobacteriaceae	Benson et al., 2010
Susceptibility to hepatocellular carcinomas	*Turicibacter*	Benson et al., 2010
Region syntenic with this associated with Crohn’s disease in humans	*Barnesiella*	Benson et al., 2010
Weight	*Akkermansia*	Davenport et al., 2015
Olfactory receptor (response to bacterial metabolites)	*Bifidobacterium*	Davenport et al., 2015
Olfactory receptor (response to bacterial metabolites)	*Faecalibacterium*	Davenport et al., 2015

The same study revealed that in the case of some taxonomic groups of bacteria, e.g., lactobacilli, host genetic control is probably exerted at the lower taxonomic ranks, i.e., at the species level and below. No QTL were identified for *Lactobacillus* (genus), so they mapped as individual traits the relative abundance of three *Lactobacillus* groups with 97% identity – *Lactobacillus reuteri, L. johnsonii*/*L. gasseri*, and *L. animalis*/*L. murinus* – to test for co-segregation at the species level. This analysis revealed that the *L. johnsonii*/*L*. *gasseri* group segregated with two significant QTL on MMU14 and MMU7 (Benson et al., 2010). Interestingly, in the case of *Helicobacter* (genus) significant QTL were detected. Both *Helicobacter* and *Lactobacillus* interact directly and adhere to host tissues, so both genera would be expected to have intrinsic susceptibility to modulation by host factors (Benson et al., 2010).

As reported by Leamy et al. (2014), a study of *G*_10_ mouse population (continued) revealed 42 microbiota-specific QTL in 27 different genomic regions that affected the relative abundances of 39 (out of the 203, i.e., 19%) microbial taxa in the murine gut. When using a strict approach the authors proposed 20 QTL as underlying genetic variation affecting the microbiota composition. The lowest FDR (false discovery rate) values, which means the greatest support, were for QTL on MMU9 affecting *Alistipes* (Leamy et al., 2014), that have been so far reported as over-represented in patients with depression (Jiang et al., 2015).

It is not clear what is the strength of these genome-related effects on microbiomes. As previously mentioned, many other factors (diet, life style, colonization order, etc.) contribute to the resultant effect. The group of Turnbaugh reported that diet dominated host genotype in shaping the gut microbiome. In mice deficient for genes linked to host-microbial interactions [MyD88(-/-), NOD2(-/-), ob/ob, and Rag1(-/-)] and in wild-type mice, gut microbiota were similarly modified by the diet. Further, the structural changes in the microbial community that were observed after dietary changes were rapid, reproducible, and reversible, thus implying the predominant role of the diet in shaping the gut microbiome (Carmody et al., 2015). Other studies dedicated to phylotypes associated with obesity, suggested that host genetic factors influenced gut microbiota plasticity in response to diet (Parks et al., 2013); this emphasizes the multilateral dependencies between various factors engaged in microbiome forming processes.

## Mechanisms that Link Host Genetics and Gut Microbiota Composition

In spite of the growing volume of data explaining how the gut microbiota affects host physiology and health, explanations of how host genetics shapes the structure of the gut microbiome are very scarce. In general, the authors propose immune functions, metabolism, energy regulation, gut motility, and adhesion interactions as the most expected genetics-dependent physiological phenomena that may impact the gut microbiota (Benson et al., 2010; Leamy et al., 2014; Davenport et al., 2015). A survey of the possible mechanisms allowing host genetics to shape microbiota composition in the gut demonstrated the major role of metabolic functions and the immune system (**Table 1**).

Benson et al. (2010) pointed out that QTL for *Coriobacteriaceae* and *Lactococcus* (located on MMU10) identified in their study were closely positioned with several genes engaged in immune responses and regulation. These comprised the TLR2 pathway, IFN-gamma, and IL-22, important in the immune response in mucosal surfaces. The authors also discussed a microbiome-related QTL on MMU1 that overlaps the conserved gene ATG16L, and the region is syntenic with a region of human chromosome 2 already shown to be associated with Crohn’s disease (Parkes et al., 2007; Benson et al., 2010). The pathogenesis of Crohn’s disease has been so far recognized as a result of the gut microbiome and environmental factors leading to an abnormal immune response in a genetically predisposed patient. Possible factors promoting and mitigating Crohn’s disease have been recently discussed in an extensive review by Manuc et al. (2016). Interestingly, some Crohn’s associated gene polymorphisms have been demonstrated as affecting both the immune response and the microbiota. For instance, the innate immune response is affected by the polymorphism of nucleotide-binding oligomerization domain-containing protein 2 (NOD2)/caspase recruitment domain-containing protein 15 (CARD15); NOD2 acts through the NF-kB pathway, which is also responsive to bacterial wall components (muramyl dipeptide). An inadequate antibacterial response related to NOD2 has been linked to probably lower production of alpha defensins (antimicrobial peptides) by Paneth cells or by an incorrect autophagy cascade with increased levels of NF-kB. It is associated with the highest risk of ileal involvement, stenoses or fistulas in Crohn’s disease (Adler et al., 2011; Lee and Lee, 2014; Strober et al., 2014; Manuc et al., 2016). Genetic variants that may also lead to an increased risk of Crohn’s disease are linked to Toll-like receptor 4 (TLR-4), typically responsible for recognizing bacterial lipopolysaccharide, CARD9 (caspase recruitment domain-containing protein 9), engaged in defense against pathogens such as yeasts, and interleukin 23 receptor (IL-23R), while IL-23 has been implicated in inhibiting the development of regulatory T cell development in the intestine (Liu and Anderson, 2014; Manuc et al., 2016). Khachatryan et al. (2008) demonstrated significant changes in microbiome structure in patients with an autoinflammatory disorder called familial Mediterranean fever (related to mutations in the MEFV gene). Their microbiome was substantially disturbed even during remissions.

The relations between gut bacteria and the central nervous system (the brain-gut axis) also engage immune system and endocrine elements. Stress has been demonstrated in rodents as the altering factor for gut microbiota through immune-activation, probably due to changes in bacterial translocation and resulting increase in stimulation of the innate immune system (O’Mahony et al., 2009; Bailey et al., 2011). As pointed out by Cryan and Dinan (2012) the mechanisms underlying this relation engage autonomic nervous system (ANS) and hypothalamus-pituitary-adrenal (HPA) axis that can modulate gut motility, secretion and epithelial permeability which impacts the niche environment for microbiota. CNS may induce signals for neurons, immune cells and others secretory cells in the gut that release a variety of signaling molecules as well as anti-microbial peptides (AMPs) and modify composition of gut microbiota (Rhee et al., 2009; Cryan and Dinan, 2012; Wang and Kasper, 2014).

Some of the identified microbiome-related QTL (on MMU7 and MMU10, for *Turicibacter* and *Coriobacteriaceae*, respectively) overlapped with QTL for murine susceptibility to carcinomas and tumor development (Benson et al., 2010). In these cases the possible role of immunological functions has not been explained yet, although tumor development usually involves complex immunological processes.

Other observations suggest that gene-encoded metabolic characteristics influence microbiome structure. For example, an correlation has been identified between a bacterial taxon associated with obesity (genus *Akkermansia*) and a variant near PLD1, a gene related to body mass index (Everard et al., 2013; Davenport et al., 2015). Evolutionary studies of vertebrates and typical composition of their gut microflora suggest that the microbiome is shaped by stomach acidity; this was confirmed by the analysis of microbiome modifications correlating with evolutionary changes of animals (Beasley et al., 2015).

Gene set enrichment analysis (GSEA) performed by Davenport et al. (2015) revealed the olfactory receptor activity significant for five taxons (family *Succinivibrionaceae*, genus *Bifidobacterium*, order *Rhizobiales*, genus *Anaerofilum*, genus *Faecalibacterium*). It had been previously demonstrated in mice that in the kidneys an olfactory receptor responded to metabolites produced by gut bacteria; this process affects renin production and it results in systemic modifications of blood pressure (Pluznick et al., 2013). Davenport et al. (2015) proposed that olfactory receptors that may be expressed in other tissues can also recognize compounds secreted by the microbiota. These olfactory receptors could play a role in host-driven regulation either for host physiology or for the microbiota in response to the gut environment.

## Discussion

In this review we present reports that suggest that the host genetic profile may shape the gut microbiome of the host. Some studies were contradictory to this statement, such as the first twin pairs analyses (Turnbaugh et al., 2009), but others revealed a possible relation between host genetics and the microbiota in the gut. A very interesting hypothesis was presented by Murphy et al. (2015) basing on results in a group of dichorionic triplet set that contained a pair of monozygotic twins and a fraternal sibling. Host genetics seems to play a role in the composition of an individual’s gut microbiome at the initial stage of life (1 month), but later (12 months) environmental factors become a major determinant.

It should be emphasized that the volume of relevant data is still small, which results mainly from technical difficulties and limitations that have also been pointed out by some researchers (Fu et al., 2016). Changes in gut microbe composition are typically analyzed quantitatively in well-represented bacterial taxa. The question that is still to be addressed relates to some small microbial groups. Possibly their quantitatively insignificant changes may affect host health substantially. Gut microbiota composition can vary significantly even in well-controlled cohorts, since other factors (than host genetics) shape the microbiome (Parks et al., 2013). A representative example is the diet, which has been demonstrated in a knock-out mice model as dominating the host genotype in shaping the gut microbiome (Carmody et al., 2015). As a result, the potential role of the low-represented microbial groups still remains vague.

A limitation comes from the relatively small size of the samples, which in microbiome studies have usually been approximately 1–2 hundred individuals, or less. The sample sizes necessary to detect significant associations in many GWAS of common diseases were thousands to tens of thousands of individuals (Davenport et al., 2015). Additionally, as pointed out by Davenport et al. (2015), at the current state of research, published replication cohorts are not available in humans. The prospects for expanding the volume of available data rely on the initiative of the Human Microbiome Project^1^, inspired by the enhancing need for understanding reciprocal cross-talk between microbiome and host. One of its major goals is to determine whether there is an identifiable ‘core microbiome’ of shared organisms, genes, or functional capabilities found in a given body habitat of all or the vast majority of humans (Turnbaugh et al., 2007).

Probably the biggest challenge that remains in the field is to answer the question: “Is it really the microbiome composition that causes a health disorder, or do both the health disorder and the altered microbiome composition result from the same genomic factor?” Probably both types of relations are possible, but as yet they have not been clearly discriminated in humans. It is very likely that a disease causes microbiome changes, due to effects on several factors that affect microbiota: changes in intestinal motility, change in appetite and diet, medical treatment including surgery, changes in lifestyle. All recent studies of Crohn’s disease confirm that it correlates with dysbiosis. Particularly, low levels of *Firmicutes, Bifidobacteria*, and *Lactobacilli* have been observed, and higher numbers of *Escherichia coli* and other strains of *Enterobacteriaceae* (Manuc et al., 2016). However, the use of probiotic bacteria such as *Lactobacillus* and *Bifidobacterium* in active Crohn’s disease or during remissions did not result in clearly conclusive, positive results. Currently, probiotics are not recommended in Crohn’s disease (Manuc et al., 2016). This example suggests that, at least in some cases, the correlation of microbiota composition and disease phenotype may result from the same detail of the host genetic profile, and they may not necessarily be directly linked.

On the other hand, there are reports demonstrating transfer of the gut microbiota between mice as sufficient for the transfer of a disease phenotype. For instance, a very recent study by Gacias et al. (2016) revealed that depression-like disorders of social behavior could be “transferred” between two genetically distinct strains of mice by the transfer of fecal bacteria. Intestinal microbes, including members of *Clostridiales, Lachnospiraceae*, and *Ruminococcaceae*, were transferred from the gut of depressed mice to those exhibiting non-depressed behavior; this was sufficient to induce social avoidance in the animals. The mechanism of this effect, as identified by metabolomics analysis, was related to increased cresol (a metabolite) levels in mice with the depressive phenotype (Gacias et al., 2016).

We propose that both types of relations between host genetic profile, gut microbiome composition, and host health are possible, thus a better understanding may allow for patient-tailored shaping of microbiome, e.g., by the diet. The field of microbiome research is anticipated to expand with new knowledge and clinical potential (Linares et al., 2016). This idea remains a fascinating area for future inquiry.

## Author Contributions

KD analyzed the literature within the topic and wrote the manuscript. WW has reviewed and consulted the manuscript.

## Conflict of Interest Statement

The authors declare that the research was conducted in the absence of any commercial or financial relationships that could be construed as a potential conflict of interest.

## Footnotes

1 http://hmpdacc.org/
